# Allergenicity Assessment of *Allium sativum* Leaf Agglutinin, a Potential Candidate Protein for Developing Sap Sucking Insect Resistant Food Crops

**DOI:** 10.1371/journal.pone.0027716

**Published:** 2011-11-16

**Authors:** Hossain Ali Mondal, Dipankar Chakraborti, Pralay Majumder, Pampa Roy, Amit Roy, Swati Gupta Bhattacharya, Sampa Das

**Affiliations:** 1 Division of Plant Biology, Bose Institute, Kolkata, West Bengal, India; 2 Post Graduate Department of Biotechnology, St. Xavier's College, Kolkata, West Bengal, India; 3 Division of Plant Biology, Bose Institute, Kolkata, West Bengal, India; Ghent University, Belgium

## Abstract

**Background:**

Mannose-binding *Allium sativum* leaf agglutinin (ASAL) is highly antinutritional and toxic to various phloem-feeding hemipteran insects. ASAL has been expressed in a number of agriculturally important crops to develop resistance against those insects. Awareness of the safety aspect of ASAL is absolutely essential for developing ASAL transgenic plants.

**Methodology/Principal Findings:**

Following the guidelines framed by the Food and Agriculture Organization/World Health Organization, the source of the gene, its sequence homology with potent allergens, clinical tests on mammalian systems, and the pepsin resistance and thermostability of the protein were considered to address the issue. No significant homology to the ASAL sequence was detected when compared to known allergenic proteins. The ELISA of blood sera collected from known allergy patients also failed to show significant evidence of cross-reactivity. *In vitro* and *in vivo* assays both indicated the digestibility of ASAL in the presence of pepsin in a minimum time period.

**Conclusions/Significance:**

With these experiments, we concluded that ASAL does not possess any apparent features of an allergen. This is the first report regarding the monitoring of the allergenicity of any mannose-binding monocot lectin having insecticidal efficacy against hemipteran insects.

## Introduction

Lectins are a group of carbohydrate-binding proteins. Many plants produce lectins as storage proteins, which also serve as defense proteins against many antagonists such as viruses, fungi, bacteria, insects and mites [1]–[6]. The insecticidal activity of plant lectins against a large array of insect species belonging to the Coleoptera, Hemiptera, Diptera and Lepidoptera order has been well documented [6], [7]. Lectins bind to glycoproteins in the peritrophic matrix or other membranous lining of the insect midgut to disrupt digestive processes and nutrient assimilation. This feature suggests a potential use of plant lectins as a naturally occurring insecticide against a number of harmful pests. Different lectins have been isolated and characterized by various groups from snowdrop, pea, wheat, rice, castor, soybean, mungbean and garlic. Some lectins, including *Galanthus nivalis* agglutinin (GNA) [8], [9], wheat germ agglutinin (WGA) [10] and concanavalin A (ConA) [11], have been reported to have detrimental effects on the sucking type of hemipteran pests.

With this unique anti-insecticidal property, some plant lectins are potential candidates for the engineering of plants with insect resistance. A number of hemipteran-specific insecticidal lectins from the GNA-related Monocot Mannose Binding Lectin (MMBL) superfamily were identified and characterized from different species of Alliaceae [2], [4], [5], [12], [13] and Araceae [13], [14]. Among them, an ∼25-kDa homodimeric lectin, *Allium sativum* (Alliaceae) leaf agglutinin (ASAL, Accession No. AY866499), interferes with the development and survival of a number of hemipteran insects, such as the rice brown plant hopper and green leaf hopper, the mustard aphid, and the chickpea aphid etc. ASAL is expressed in a number of agriculturally important crops such as rice [4], mustard [15] tobacco [2], [16] and chickpea [3], which exhibit significant levels of resistance against the above-mentioned pests. Each subunit of the homodimeric ASAL bears three potential mannose-binding motifs consisting of the following five amino acid residues: Gln, Asp, Asn, Val and Tyr (QDNVY). These five residues comprising the polar surface of the binding pockets are completely conserved throughout the MMBL superfamily [17]. In all studied structures of this lectin superfamily [18], the subunits assemble into a stable dimer by exchanging their C terminal β-strands to form a hybrid β-sheet [19], which is crucial for its insecticidal activity.

**Figure 1:**
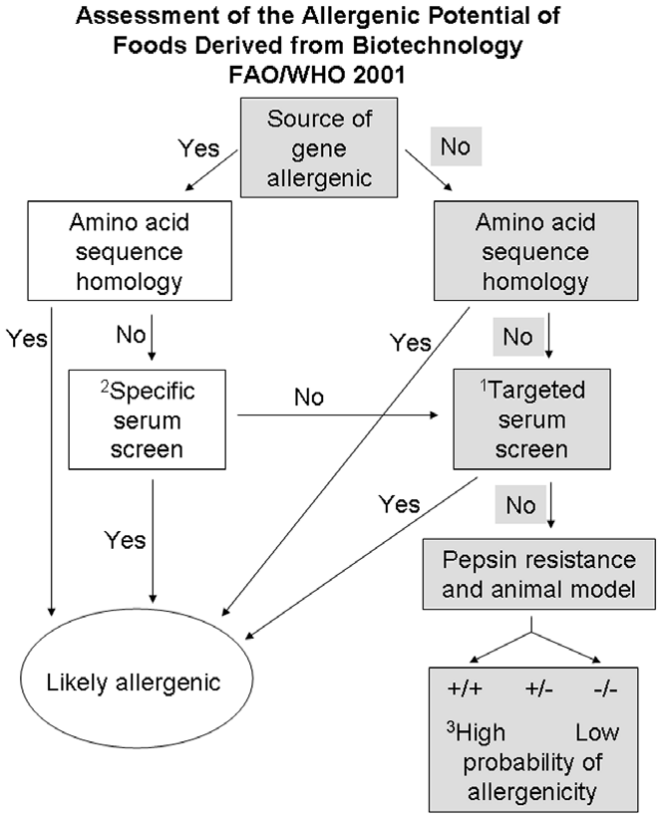
FAO/WHO 2001 decision tree (reproduced from http://www.fao.org/docrep/007/y0820e/y0820e00.htm). The approach used in the present study is shown in gray. ^1^Screening of serum samples from population allergic to the food group. ^2^IgE binding to test protein from sera of individuals with known allergies to the source of the novel protein. ^3^When positive results are obtained in both the pepsin resistance and animal model protocols, the expressed protein has a high probability to become an allergen. When negative results are obtained in both protocols, the expressed protein is unlikely to become an allergen. When different results are obtained in the pepsin resistance and animal model protocols, the probability of allergenicity is intermediate.

Nevertheless, there is a growing concern among the scientific community as well as among laypeople regarding the potential risk of the use of genetically modified food crops on the health of consumers and non-target organisms. It is highly recommended to perform the *in vitro* safety assessment study of the gene to be used in transgenic plant development program. The reliability of the safety assessment strongly depends on the monitoring of any allergic reactions triggered by the gene products. The joint consultation of the Food and Agriculture Organization (FAO) and World Health Organization (WHO) held in Rome, Italy on 25th January, 2001, focused on the safety aspects of genetically modified foods and discussed the issue of allergenicity of genetically modified foods [20]. Arising out of the 2001 Joint FAO/WHO Consultation, a new decision tree for assessing the allergenic potential of a protein of interest has been proposed (**Figure 1**). As a result of this meeting, all biotechnologically significant proteins must be monitored by the following guidelines [20]: (a) by determining the allergenic/non-allergenic source of the gene, (b) by analyzing sequence homology to reported allergens (food and environmental), (c) by serum screening for cross-reactivity with sera from patients who are allergic to materials that are broadly related to the source material of the protein, (d) by assessing the pepsin resistance of the gene product and (e) by monitoring digestibility of the protein in animal models. This decision tree process was published [21] and subsequently accepted by biotechnological crop industries and regulatory agencies.

The allergic reaction usually occurs in the gastrointestinal tract (nausea, vomiting, diarrhea); skin (urticaria, dermatitis, angioedema); and respiratory tract (rhinitis, asthma, bronchospasm). The food allergy is usually mediated by Immunoglobulin E (IgE). The gastrointestinal tract mucosa of all organisms is composed of glycoproteins, which have an affinity for carbohydrate-binding proteins through their mono- or oligosaccharide moieties. Many lectins fall under this category. Seeds from a number of leguminous plants, rich in lectins and major constituents of our daily food intake, are allergenic to a significant fraction of the human population [22]. Knowledge about the physio-chemical properties of plant lectins and the effects on animals and humans has been generated from feeding experiments with certain lectins, particularly phytohaemagglutinin [23], concanavalin A [24], and *A. sativum* bulb lectins (ASA I and ASA II) [25]. A few reports on hypersensitivity to garlic (*A. sativum* bulb) are available as contact dermatitis, rhinoconjuctivitis, asthma and urticaria [26], [27], but there is no report on the allergenicity of the garlic leaf, which is the source of ASAL.

According to the recommendation of the Joint FAO/WHO Consultation (2001), the present study was framed (**Figure 1**) to explore the allergenicity of a biotechnologically significant insecticidal lectin, ASAL. The sera from common allergy patients were assessed through an IgE-mediated hypersensitivity reaction experiment. The study was extended by analyzing the sequence homology to known allergens. Furthermore, the digestion of ASAL was performed in simulating gastrointestinal fluid (SGF) [28]. Feeding assays with purified ASAL in mice were monitored to assess the stability of ASAL in response to digestive enzymes *in vivo.*

## Materials and Methods

### Animal ethics statement

Albino mice were collected from the vendor with necessary approval of the Ethics Committee of the Bose Institute and used for an *in vivo* digestion stability assay under the permit number AEC/BI/SD/PS/1/2010.

### Human ethics statement

Approval (Ref no. ICH/Sys-5/085/2010) was obtained from the Medical Officer-In-Charge, Allergy Department, on behalf of the Ethics Committee, Institute of Child Health, Kolkata, India to collect the blood sera of allergic and healthy human subjects and to perform necessary tests. All participants provided written informed consent.

### Consent from all authors involved in the study

The manuscript was prepared and submitted as all participants provided written informed consent.

### Materials

Fresh garlic (*Allium sativum* L.) leaves were collected from the plants grown in the institutional experimental farm.

### Patients

Two hundred and sixteen allergic patients (mean age 33.4±1.2 years; male:female, 7∶10) were selected from the outpatient clinic of the Allergy Department, Institute of Child Health, Kolkata, India on the basis of clinical history and diagnosis. The patients were allergic to foods of plant origin, such as brinjal, tomato, spinach, drumstick, pumpkin, and okra, and each of them reported that his or her regular diet contained garlic. Twenty five non-atopic volunteers (mean age 32.8±0.9 years; male:female, 7∶9) were selected as the control group. The exclusion criteria were perennial or severe asthma, pregnancy or lactation, and malignancy or other systemic diseases during sera collection. To avoid the masking of possible symptoms, the use of corticosteroids and antihistamines was prohibited.

### Analysis of purified ASAL by MALDI-TOF mass spectrometry

ASAL was purified through affinity followed by ion-exchange chromatography as described in previous report [12]. The purified ASAL was analyzed in 15% SDS-PAGE, subsequently dry droplet method was used to crystallize the protein sample. One microliter of prepared sample was mixed with 1 µl sinapinic acid (SA) matrix. Before using the matrix, the SA was sonicated for 10 min. Then, 1 µl was taken from the saturated supernatant. The sample mix (2 µl) was loaded on to the 384-well MALDI target steel plate (Bruker Daltonik, GmbH) and then dried at room temperature to form crystals. The protein mass fingerprinting (PMF) of ASAL was determined by MALDI-TOF mass spectrometry in an Autoflex II MALDI-TOF/TOF mass spectrometer (Bruker Daltonik, GmbH) in linear mode with SA as the matrix. Mass spectral data were obtained with a 337-nm N_2_ laser at 54% power in the positive ion mode. The final data were obtained by averaging 100 spectra, each of which was the composite of 20 laser firings. The spectra were processed using Flexanalysis 2.4 software (Bruker Daltonik, GmbH).

### Bioinformatics Study

An efficient and comprehensive web tool, Allermatch™ developed by Fiers et al. 2004 [29] was used to analyze the potential allergenicity of the ASAL sequence according to the current FAO/WHO Codex alimentarius guidelines. Allermatch™ provided two search methods according to the FAO/WHO guidelines, and a third method was also provided as an extra tool. The first mode was the sliding window approach that divided the query protein sequence into an 80-amino acids window using a sliding window with a shift of a single residue. Each of these windows was compared with all sequences in the allergen database of choice. In the second mode (word match), the software looked for short sub-sequences of default 6 amino acids (words), which had a perfect identity with a database entry. The third mode was the full FASTA alignment with an Allermatch™ allergen database.

### IgE-Specific ELISA

Sera were collected from each of 216 allergic patients and 25 non-allergic volunteers. An ELISA was performed to determine ASAL-specific IgE levels in individual sera using an antihuman IgE horseradish peroxidase conjugate (Sigma-Aldrich, St Louis, MO, USA) in a 1∶1200 dilution and o-phenylene diamine substrate [30]. The absorbance was measured with an ELISA reader (BIO-RAD model 680) at 492 nm. For individual patient serum, the P/N value (ratio of O.D. of individual patient sera with respect to the control group) was determined [31]. Here, the control was the mean O.D. value from the panel of 25 healthy volunteers' sera.

### Pepsin Digestion Assay

The pepsin digestion protocol described by Astwood et al. 1996 [32] was followed. The simulated gastric fluid (SGF) reaction buffer was prepared by adding 122.8 mg of NaCl to 59.2 ml of distilled water and adjusting the pH to 1.2 using 6 M HCl. The amount of pepsin (Sigma) used to prepare SGF was calculated from the specific activity of the product. For digestion purposes, the ASAL was concentrated in a Microcon® centrifugal filter device (Milipore) in 35 mM NaCl, pH 7.5. The assay was designed for fixed volumes and a fixed amount of test protein as described by Astwood et al. 1996 [32] and Fu et al. 2002 [33]. Two sets of ASAL and pepsin were used that were equivalent to 45.6 and 10 units of pepsin activity per microgram of ASAL, respectively. ASAL was added to each SGF reaction vial to start the digestion by maintaining the above criteria. After periods of 0, 2, 5, 15, 30, 60 and 120 min, 0.5 µl of 5 N NaOH was added to stop the reaction. Immediately after stopping the reaction, Laemmli buffer [34] was added to each vial prior to heating for 4 min in a boiling water bath. Then, each sample was loaded onto a 15% reducing SDS-PAGE along with protein molecular weight markers. The gel was run at a constant voltage, and protein bands were visualized by Coomassie brilliant blue staining.

### Western blot assay of pepsin-treated ASAL

Pepsin-treated ASAL (1 µg ASAL:10 units pepsin) from different time points was resolved by 15% SDS-PAGE, including untreated ASAL as a positive control. The gel was subsequently electroblotted to a nitrocellulose membrane (Amersham Biosciences) with a constant 200 mA current for 1 h (14). First, incubation was performed with an anti-ASAL polyclonal antibody (raised in rabbit by the present group) in a 1∶8000 dilution. Then, the membrane was incubated with an anti-rabbit IgG (raised in goat, Sigma) horseradish peroxidase conjugate at 1∶20000 for probing the treated and untreated ASAL electroblotted on the nitrocellulose membrane.

### Thermal stability Assay

The stability of the protein was assessed by its ability to agglutinate rabbit erythrocytes. Rabbit blood was diluted in 0.9% NaCl to a final stock solution concentration of ∼5% erythrocytes. The working concentration of rabbit erythrocytes was ∼1.5%. The minimum concentration of ASAL to agglutinate the rabbit erythrocytes was identified prior to assessing the stability of ASAL. Thus, 0.625 µg of ASAL was used in phosphate buffered saline (PBS), and incubated separately at 25, 37, 55, 75 and 95°C for 30 min. Upon incubation, each sample was subjected to rapid cooling on ice, and agglutination activity was observed. Furthermore, to obtain the exact temperature of stability, the same concentrations of ASAL were incubated to 40, 45, 50 and 55°C, and the subsequent agglutination activities were observed.

### ELISA of fecal matter

Nine albino mice of ∼150 g each were divided into two groups. Three mice constituted the control group while six others were used for the ASAL feeding group. All of the mice were housed in a room with controlled temperature (22±2°C), humidity (55±5%) and a 12:12 h light: dark cycle. Mice were maintained on a commercially available mannose-free normal pellet diet and water ad libitum for a week to acclimatize to lab conditions. After acclimatization, they were kept for 24 h without food but with water. After that time period, they were fed with a diet soaked in 50 mg purified ASAL dissolved in 20 mM Tris-Cl (pH 7.4). For the control group, mice were fed with same amount of diet soaked in 20 mM Tris-Cl (pH 7.4). After feeding, fecal matter was collected for up to 24 h for an ELISA. Then, the mice were dissected, and the small and large intestines were separated.

The fecal matter of ASAL-fed and control-fed mice was collected separately and then soaked and suspended in coating buffer overnight at 4°C. After vigorous shaking by a vortex for 5 min, the samples were centrifuged at 2000 rpm for 5 min at 4°C to eliminate the insoluble fraction. Two hundred microliter of the fecal suspension was then loaded into the wells of a microtiter plate and kept overnight at 4°C. The wells of the microtiter plate also contained 200 µl of serially diluted pure ASAL in coating buffer. Five percent nonfat milk (Merck) in phosphate buffered saline containing Tween-20 (PBST) was used to block the wells at 37°C for 2 h. Two hundred microliter of a 1∶4000 diluted anti-ASAL serum was added to each well and incubated at 37°C for 1 h. Two hundred microliters of horse radish peroxidase (HRP)-conjugated anti-rabbit IgG at a 1∶4000 dilution was used as the secondary antibody and incubated at 37°C for 1 h. The color was developed using 9 mg O-Phynelene diamine (OPD) dissolved in 20 ml citrate buffer (0.1 M citric acid and 0.1 M sodium citrate mixed to pH 5.0) at room temperature for 20 min in the dark. The reading was acquired on a microplate reader at 415 nm wavelength.

### Immunohistolocalization of the Intestine in mice

After dissecting both control and ASAL-fed mice, both small and large intestines were cut into small pieces and fixed with 1% glutaraldehyde and 4% paraformaldehyde in 50 mM PBS, pH 7.4, overnight. For the small intestine, both the duodenum and ileum were removed. Transverse sections of the gut were collected using a Leitz cryostat 1720 at 25°C. The sections were then washed with PBST at room temperature and blocked with 5% nonfat milk in PBST for 2 h at 37°C and were then incubated for 1 h at 37°C with anti-ASAL serum at 1∶1000 dilution in PBST. Anti-rabbit IgG with an Alkaline phosphatase conjugate was then incubated with the sections at a 1∶2000 dilution in PBST at 37°C. The color was developed by adding Nitro Blue Tetrazolium/5-Bromo-4-Chloro-3-Indolyl Phosphate (NBT/BCIP) substrate.

### Western blot analysis of the intestine of ASAL-treated mice

Different parts of the mouse intestine were washed and kept in PBS with 0.02 M poly methyl sulfonyl floride (PMSF) and then crushed in a homogenizer. The samples were centrifuged to discard the debris, and the clear supernatant was collected. The supernatant was then subjected to 15% SDS-PAGE analysis, and the western blot was developed as described above using an ECL chemiluminescence kit (Amersham Biosciences) on KODAK X-ray film.

## Results

### Analysis of purified ASAL by MALDI-TOF mass spectrometry

**Figure 2:**
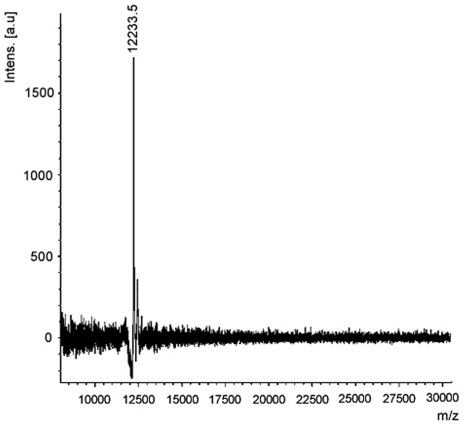
MALDI-TOF mass spectrometry of ASAL. This profile illustrates intact peptide mass that is typical for the mass spectra of ∼12.2 kDa. Appearance of one peak of ∼12.2 kDa confirms the quality of purification as well as its homodimeric nature of native ASAL

ASAL that was purified through affinity chromatography followed by ion-exchange chromatography was subjected to 15% SDS-PAGE analysis (data not shown), which showed single bands of ∼12.2 kDa [12]. The MALDI-TOF profile authenticated the purity of the ASAL (**Figure 2**).

### Bioinformatics study

The Allermatch™ web tool [28] was used to test the ASAL (Accession No. EU252577, amino acid sequence: MARNLLTNGEGLYAGQSLDVEQYKFIMQDDCNLVLYEYSTPIWASNTGVTGKNGCRAVMQRDGNFVVYDVNGRPVWASNSVRGNGNYILVLQKDRNVVIYGSDIWSTGTYRR). Through the sliding window approach, the ASAL primary amino acid sequence (112 amino acids) was divided into 80 amino acid-containing fragments, and 33 windows were analyzed (112-80+1 = 33) with steps of a single residue (amino acid) with a default setting of 35% cut-off and a six-word match at a time. After the analysis of the primary amino acid sequence of ASAL, no significant matches (35% homology or at a stretch of six amino acids) were observed with any of the known allergens of either the Swiss-Prot or the WHO-International Union of Immunological Societies (IUIS) database. Using a setting of a 29% cut-off value or above, no allergens from Swiss-Prot or WHO-IUIS were matched to the ASAL sequence. When an exact hit of six amino acids in a sequence in the databases [SwisProt and WHO-IUIS] by analysis of 107 windows (112-6+1 = 107) was searched, no significant matches were found. We also extended our effort using an Allermatch™ analysis tool to look at the ASAL sequence for a full FASTA alignment, although full alignment was not required according to the FAO/WHO Alimentarius guidelines [20]. The highest percentage of identity was obtained at 22.5 with two allergens (Peptidase 1 of the American house dust mite and polygalacturonase of timothy grass) from the WHO-IUIS and Swiss-Prot databases.

### IgE-Specific ELISA

An ELISA test cannot predict the severity of an allergic reaction, but it can evaluate the IgE binding potential of certain proteins. The P/N value for ASAL did not exceed 1.25 (**Table 1**). With such a low P/N value, ASAL is not considered to be an allergen [31]. Altogether, the P/N values were found to be in the range of 0.9-1.25.

**Table 1:** Table showing the *in vitro* IgE specific ELISA results.

Range of P/N* Value	Number of Patient Serum Sample
0.9-0.95	21
>0.95-1.00	34
>1.00-1.05	49
>1.05-1.10	50
>1.10-1.15	26
>1.15-1.20	28
>1.20-1.25	08

*IgE-reactive proteins shows P/N value > 3.5 [31].

### Pepsin digestion assay

**Figure 3:**
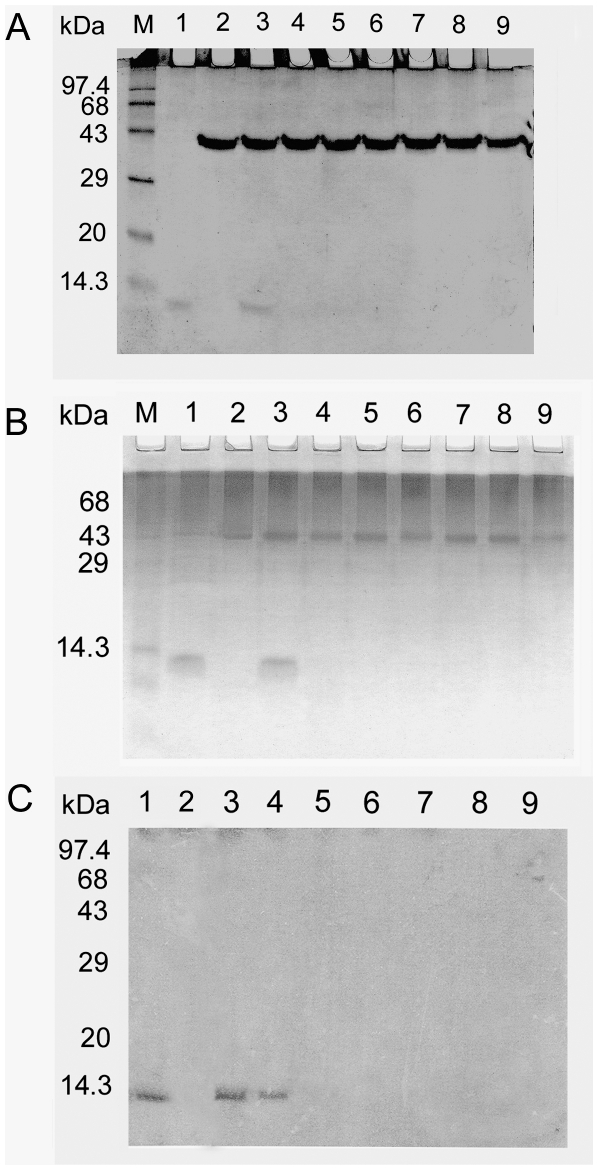
SDS-PAGE analysis of pepsin treated ASAL. (A) Lane M: Molecular weight marker; lane 1: ASAL (∼1 µg); Lane 2: pepsin (45.6 units) in SGF; Lanes 3 to 9: Incubation of ASAL with pepsin amended SGF for 0, 2, 5, 15, 30, 60 and 120 min. (B) Lane M: Molecular weight markers; Lane 1: ASAL (∼1 µg); Lane 2: pepsin (10 units) in SGF; Lanes 3 to 9: Incubation of ASAL with SGF for 0, 2, 5, 15, 30, 60 and 120 min. (C) Western Blot analysis of the degradation of ASAL in SGF. Lane 1: ASAL as positive control; Lane 2: SGF preparation only; Lanes 3 to 9: Incubation of ASAL with SGF for 0, 2, 5, 15, 30, 60, 120 min.

The pepsin digestibility assay was used to determine the relative stability of ASAL. ASAL was not detected by SDS-PAGE and Coomassie brilliant blue staining after 2 min of incubation in pepsin-amended SGF in 1 µg ASAL with 45.6 units of pepsin (**Figure 3A**) and in 1 µg ASAL with 10 units of pepsin (**Figure 3B**). A western blot assay (1 µg ASAL with 10 units of pepsin) to detect the digestion profile after different time points showed no bands after 2 min of ASAL incubation with pepsin (**Figure 3C**).

### Thermal stability assay

**Figure 4:**
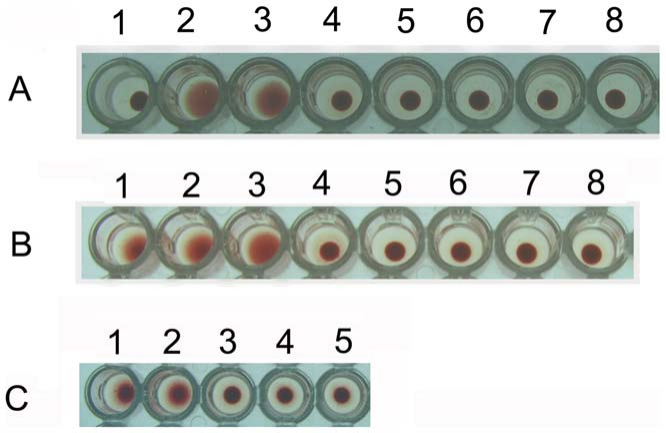
Thermal Stability Assay of ASAL. (A) Determination of minimal dose of ASAL required to agglutinate the rabbit erythrocytes. No. 1: Control (100 µl of 1.5% rabbit erythrocytes incubated without ASAL); No. 2 to 8: Incubation of prepared rabbit erythrocytes with 1.25, 0.625, 0.312, 0.156, 0.08, 0.04, 0.02 µg ASAL; 0.625 µg ASAL agglutinated 100 µl of 1.5% rabbit erythrocytes. (B) Incubation of 100 µl of 1.5% rabbit erythrocytes with ∼0.625 µg ASAL at different temperatures. No. 1: Control (100 µl of 1.5% rabbit erythrocytes with ∼0.625 µg ASAL); No. 2 to 6: ASAL treated at 25, 37, 55, 75 and 95°C for 30 min; No. 7: ASAL treated at 100°C for 5 min; No. 8: Control (rabbit erythrocytes without ASAL). ASAL lost agglutination activity upon temperature treatment at 55°C incubation for 30 min. (C) Incubation of ASAL at 37 to 55°C. No. 1 to 4: ASAL treated at 40, 45, 50 and 55°C for 30 min; No. 5**:** Control (rabbit erythrocytes without ASAL). Scattered and centrally located matters demonstrated agglutinated and non-agglutinated rabbit erythrocytes respectively.

As low as 0.625 µg ASAL was found to be essential to agglutinate 1.5% rabbit erythrocytes (**Figure 4A**). In a thermal stability experiment, ASAL was stable up to 45°C but labile at 55°C upon incubation for 30 min which resulted in loss of agglutination activity (**Figure 4B**). By optimizing the temperature across the range of 40 to 55°C, ASAL lost biological activity by a 30 min incubation at 50°C (**Figure 4C**).

### ELISA of fecal matter

After 24 h of feeding with 50 mg of purified ASAL, the fecal matter of mice was collected and analyzed for the presence of ASAL through an ELISA assay. Using different concentrations of fecal matter, the OD value for the purified ASAL-fed mice was nearly the same as the control OD (data not shown).

### Immunohistolocalization of intestine of ASAL-treated mice

**Figure 5:**
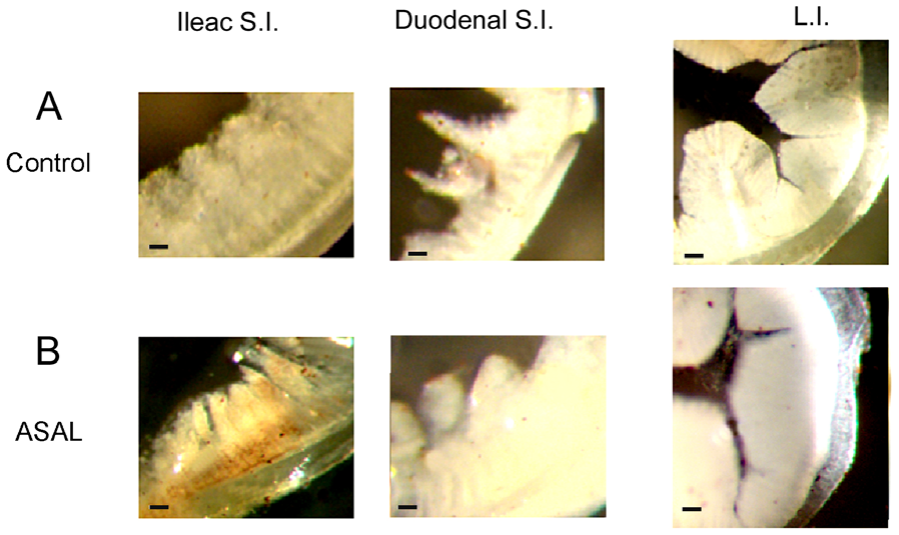
Immunohistolocalization of mouse gastrointestinal tract. Panel A: Sections of different parts of GI tract of mouse fed with only diet. Panel B: Sections of different parts of GI tract of mouse fed with ASAL supplemented diet. Left column showing small intestine (S.I.) at ileac end, middle column showing S. I. at duodenal end, right column showing sections of large intestine (L.I.). Scale bars  =  500 µm.

Various parts of the guts of ASAL-fed and control mice were collected 24 h after feeding, and an immunohistochemical assay was performed to investigate the binding of ASAL to the brush border membrane. As seen in **Figure 5**, there was very little or no difference in the color deposition at the brush border membrane of control mouse guts and the guts of ASAL-fed mice. The lack of detectable binding at the gut membrane despite the quantity of pure ASAL fed to the mice indicated its digestion.

### Western blot analysis of intestinal extracts of ASAL-treated mice

**Figure 6:**
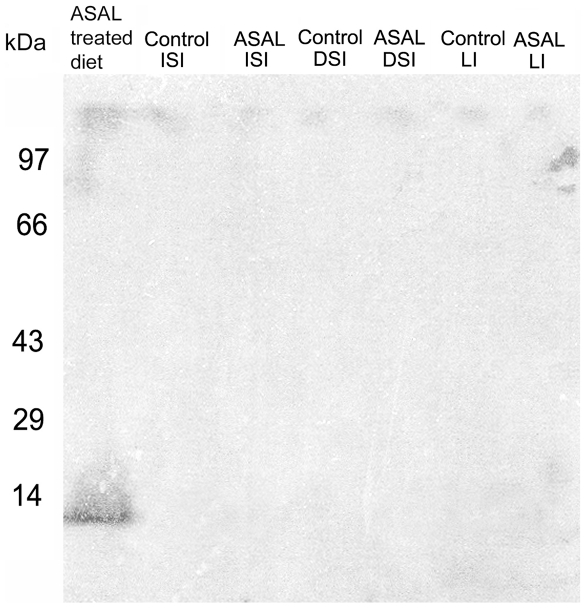
Western blot analysis of the tissue extracts of mouse gastrointestinal tract. Molecular weight markers are mentioned in kDa shown at Y axis. Lane marked ASAL loaded with purified ASAL, while the sample name is written at the top of each lane. ISI: Small Intestine from Ileac part, DSI: Small Intestine from Duodenal part, LI: Large Intestine.

Western blot analysis of tissue extracts from various regions of the intestine with an anti-ASAL antibody showed no significant signal (**Figure 6**), which further confirms the observation of the absence of ASAL binding to the brush border membrane of gut tissue.

## Discussion

Since the mid-1990s, the rapid adoption of genetically modified (GM) crops among farmers resulted from one or many beneficial characteristics such as the increase in yield potential, minimization of yield loss caused by the attack of damaging pests, minimization of production cost and improvement in quality of the crops and the food produced from them. In recent years, many transgenic crops have been released by plant biotechnological companies and research institutions. In our laboratory, ASAL has been expressed successfully in tobacco [2], rice [4], mustard [15] and chickpea [3], which demonstrated an antagonistic effect against the colonization of their respective target pests. Consequently, several questions concerning the food and environmental safety aspects of the crops in general have been raised. Considering the usual concern about the possible allergenicity of GM crops, the decision-tree approach was adopted for safety assessment.

### Source of ASAL

There is no single protocol available to judge the allergenic potential of a protein, which relies on a number of ‘weight of evidence’ approaches. The safety of the source organism is a considerable factor. A gene derived from a commonly consumed food crop does not provoke the same degree of scrutiny as would the use of gene from a highly toxic source. However, in practice, the degree of scrutiny is the same. In the present study, following the decision tree, the source of the gene was first considered (**Figure 1**). The source of ASAL is garlic leaf, of which there is no published report of allergenicity available in the current literature. Thus, the source of the gene may be considered to be non-allergenic. Then, according to the decision tree, a comparison of amino acid sequences of test proteins with known allergens, serum screening, monitoring the stability of the protein to gastric fluids (pepsin resistance) and heat and testing of digestibility in an animal model were applied as methods of assessment.

### Amino acid sequence homology

A number of major food and respiratory allergens have already been identified, and Swiss-Prot and WHO-IUIS databases have been developed. Therefore, candidate proteins can be screened for similarity to known allergens through a bioinformatic approach prior to product development [35]. Proteins sharing less than 50% identity over their entire length are unlikely to be cross-reactive, and more than 70% identity often shows as cross-reactive [36]. After full alignment, ASAL did not match any known allergen proteins above 22.5% homology. Through an 80-amino-acid, sliding window approach, ASAL did not match any allergenic proteins above a score of 29%. A recent FAO/WHO scientific panel recommended using a six-amino-acid window for this type of analysis [20]. However, Hileman et al. 2002 [37] showed that an amino acid window size of less than eight amino acids resulted in a high rate of false positives. Through an Allermatch™ analysis of six amino acids, no allergens were matched to ASAL.

### Targeted serum screening

A candidate protein cannot be ascertained as non-allergenic even if it does not show significant homology to reported allergens. Specific and targeted sera screening is necessary because IgE-mediated allergies are very common, and it is an alternative procedure to screen an *in vitro* allergenicity effect. Targeted sera screening was used in the present study because the source of the gene is non-allergenic. Through sera screening, the ASAL P/N ratios did not exceed 1.25 (**Table 1**), which is quite low compared to normal allergens. Previously, Chakraborty et al. 2005 [31] reported that IgE specific ELISA on *Carica papaya* pollen allergens challenged with patient sera followed by IgE specific secondary antibody incubation showed a P/N value > 3.5. IgE-ELISA of a protein with P/N value > 3.5 was suggested to be potentially IgE reactive [31].

### *In vitro* pepsin digestibility assay

Digestion stability for a protein is also a key prerequisite for evaluating allergenicity [21]. In order for a protein to elicit an allergic response, it must survive in the acidic and proteolytic environment of the human GI system and be absorbed through the intestinal mucosa [38]. Stability or instability of a protein in SGF depends on the relative amounts of enzyme and test protein [32], [33]. Some studies have reported comparatively low ratios (by weight) of enzyme:protein, ranging from 0.1 to 0.01 [39]; however, higher ratios ranging from 25 to 5,000 [33], [40] have also been reported. Fuchs and Astwood 1996 [41] showed that nine different non-allergenic proteins were rapidly degraded within 30 seconds in SGF compared to those designated as allergens that took more than two minutes to be degraded. Others have employed different time frames for defining the stability of a protein. Momma et al.1999 [42] demonstrated that a soybean glycinin expressed in genetically engineered rice was labile in SGF within 30 min. Noteborn et al. 1995 [40] concluded that Cry1Ab was labile to digestion in SGF, although a 15-kDa fragment was still present after 120 min of pepsin digestion. Therefore, these studies revealed the difficulty of establishing a standard guideline for the interpretation of digestion assay results. We assessed the stability of ASAL in two sets of SGF with two different enzyme:protein ratios as described by Astwood et al. 1996 [32]. Both ratios of enzyme and ASAL showed the same results, and ASAL was digested in 2 min (**Figure 3**). Generally, food allergens remain stable in SGF for more than 2 min of a pepsin-amended incubation in the same experimental conditions [32]. We further extended our efforts to detect ASAL or its stable products through western blot analysis, and no bands were detected in ASAL samples after incubation with pepsin for two minutes.

### Thermolability of ASAL

Most food allergens are proteins and generally contain intramolecular disulfide bonds. The structural conformation of a protein may be an important factor for an allergen to resist denaturation. In addition, thermal processing can create new allergic epitopes that can modify the existing epitopes [43]. Thus, whether and how heat treatments may alter the allergenicity of food is a complex question [44]. Resistance to heat denaturation is common in several food allergens; thus a correlation can easily be drawn between heat stability and allergenic potential. Thermal stability or the stability of protein during post-translational processes is part of the evidence used to assess the potential allergenicity of a particular protein. Therefore, the retention of biological activity after incubation under high temperature conditions may indicate that a protein is allergenic. In this regard, ASAL was thermolabile at 50°C upon 30 min of incubation (**Figure 4**).

Stability to gastric juices and heat are not absolute predictors of allergenicity. Many indigestible proteins in food have no history of allergenicity, and a few rapidly digestible proteins, such as patatin from potatoes, are allergens to some people. Assessment of the stability to gastric fluids and temperature provide information as to whether a protein is retained in its native form and is able to interact with the immune system. Another possibility is that glycosyl groups of a protein may contribute to its allergenicity because many allergens are glycoproteins. The glycosylation patterns may differ substantially from their native counterparts when transgenes are expressed at an abnormally high level in tissue from which they are normally absent or when two plants across a wide species barrier are crossed [45]. Although, no N-glycosylation motif (Asn-Xaa-Ser/Thr) (Accession No.EU252577), which imparts the ability to covalently attach to oligosaccharides during post-synthesis modifications, is seen in the primary amino acid sequence of ASAL. Therefore, there is only a remote possibility that the expressed ASAL is allergenic in the transgenic plants.

### Fate of ASAL when consumed by an animal model

Digestibility of a protein is dependent not only on the enzymes but also on other factors that are present in the gastrointestinal tract. Various reports state that lectins are digestible *in vitro* but not *in vivo* and vice versa. No significant trace of ASAL was recorded in the fecal matter of lectin-fed mice, which indicates the digestibility of ASAL in the *in vivo* condition. Further immunolocalization detected insignificant binding of ASAL in the mouse gut membrane (**Figure 5**). The scarcity of α-1, 3 terminal mannose residues in the brush border membrane of the small intestine of mammals may be a limiting factor here [46], although previously, GNA was shown to bind to the mouse gut [47]. However, prolonged GNA exposure of up to 10 days did not show significant changes in the gut properties of mice and was considered to be ‘non-toxic’.

### Conclusions

Both *in vitro* and *in vivo* experiments showed that ASAL was easily digested, and thus the possibility of this lectin being allergenic is very low. This result was further confirmed by *in vitro* tests that showed no IgE-mediated hypersensitivity reactions. According to the FAO/WHO decision tree, when negative results are obtained in both the pepsin digestibility assay and animal model experiments, the expressed protein is unlikely to become an allergen. Thus, ASAL can be an important component of an integrated pest management program as a safe insecticidal lectin.
